# Mass segmentation using a combined method for cancer detection

**DOI:** 10.1186/1752-0509-5-S3-S6

**Published:** 2011-12-23

**Authors:** Jun Liu, Jianxun Chen, Xiaoming Liu, Lei Chun, Jinshan Tang, Youping Deng

**Affiliations:** 1 College of Computer Science and Technology, Wuhan University of Science and Technology, Wuhan, Hubei, China; 2 Key laboratory of Molecular Biophysics of the Ministry of Education, College of Life Science and Technology, Huazhong University of Science and Technology, Wuhan, Hubei, China; 3 Rush University Cancer Center, Rush University Medical Center, Chicago, Illinois, USA

## Abstract

**Background:**

Breast cancer is one of the leading causes of cancer death for women all over the world and mammography is thought of as one of the main tools for early detection of breast cancer. In order to detect the breast cancer, computer aided technology has been introduced. In computer aided cancer detection, the detection and segmentation of mass are very important. The shape of mass can be used as one of the factors to determine whether the mass is malignant or benign. However, many of the current methods are semi-automatic. In this paper, we investigate fully automatic segmentation method.

**Results:**

In this paper, a new mass segmentation algorithm is proposed. In the proposed algorithm, a fully automatic marker-controlled watershed transform is proposed to segment the mass region roughly, and then a level set is used to refine the segmentation. For over-segmentation caused by watershed, we also investigated different noise reduction technologies. Images from DDSM were used in the experiments and the results show that the new algorithm can improve the accuracy of mass segmentation.

**Conclusions:**

The new algorithm combines the advantages of both methods. The combination of the watershed based segmentation and level set method can improve the efficiency of the segmentation. Besides, the introduction of noise reduction technologies can reduce over-segmentation.

## Background

Breast cancer is one of the leading causes of cancer death for women all over the world [1] and early detection is one of the main ways to reduce the death rate of the human beings with breast cancer [2-4]. One of the ways to detect the breast cancer is to use mammography. Mammography is thought of as one of the most effective methods to detect early breast cancer. Although mammography is widely used, the rate of correct diagnosis of breast cancer using mammography needs improvement [5]. Thus, in order to improve the diagnosis rate, computer aided diagnosis was proposed to assist the radiologists in the diagnosis of the breast cancer and used to improve the diagnosis accuracy [6].

In computer aided cancer diagnosis, the detection and segmentation of mass are very important. The shape of mass can be used as one of the factors to determine whether the mass is malignant or benign. In the past, many methods for mass segmentation algorithms have been proposed. These algorithms include manual segmentation [7], semi-automatic segmentation [8], and fully automatic segmentation [9]. Although manual segmentation is considered to be the best mass boundary extraction method [10,11], it is time-consuming. Besides, it subjects to intra-observer and inter-observer variation [11]. In [12], Huo et al. developed a semi-automatic region growing approach based on the choice of the starting point by the radiologist. In [13], Kobatake et al. applied a modified Hough transform to extract lines passing near the centre of the mass and automatically selected candidates based on the number of line-skeletons. In [14], Lou et al. proposed an algorithm for mass segmentation and the algorithm is based on the assumption that the trace of intensity values from the breast region to the air-background is a monotonic decreasing function. In [15], Zheng et al. proposed an algorithm using the difference image obtained by subtracting the Gaussian filtered image from the original image. In [16], Petrick et al. proposed a method for mass segmentation. The basic idea of the proposed method is to select seeds using local maxima in the original image and generate a gradient image using a frequency-weighted Gaussian filtering. With this image, the thresholds of the regions bounded by the edges are extracted. In [17], Qi and Snyder proposed a method for mass segmentation. They used B'ezier splines to interpolate histograms, from which they extracted the region with threshold values at local maxima. In [18], Guliato et al. proposed a pixel based algorithm. The proposed algorithm aims to preserve the transition between masses and normal tissue to segment the mass boundary. In [19], Mudigonda et al. used multilevel thresholding to detect closed edges for mass segmentation. Besides the work mentioned above, there is also other work published in [20-22].

Although many other results on mass segmentation have been published, automatic segmentation of mass is still considered difficult because of the ill-defined boundaries and overlapping with fibro-glandular tissue of many masses [11]. In this paper, we study fully automatic mass segmentation algorithm. Our basic idea is to combine two segmentation algorithms: watershed based segmentation algorithm and level set based segmentation, As is well known, level set based segmentation methods are powerful image segmentation tools and have been used for image segmentation for long time because they have many advantages, for examples, they can handle any of the concavities, splitting, merging and so on. Thus they are still used in many fields including medical image processing [23]. However, there are several disadvantages on level set based segmentation methods. One of the main disadvantages is that the computation is costive. Besides, the level set based algorithms generally need human interaction. In order to reduce the interaction, this paper proposes an algorithm which combines a fully automatic marker-controlled watershed segmentation method with level set based segmentation. In the combined algorithm, the segmentation results from the watershed are used as the input of the level set segmentation and the level set algorithm is used to refine the boundary.

## Results

### Experimental materials

In the experiments, we selected 200 mammograms randomly from the DDSM database [24] to verify the proposed algorithm. For reducing computation cost, we resample the original images at a reduced pixel size and 256 gray levels. The mass location was identified by an experienced radiologist and a region of interest (ROI) containing the mass was extracted. The selected samples contain lesions with different breast-tissue density, different degrees of subtlety, and different sizes. The distributions of the size of malignant and benign masses overlapped. 100 of the dataset are benign and 100 of them are malignant.

**Figure 1:**
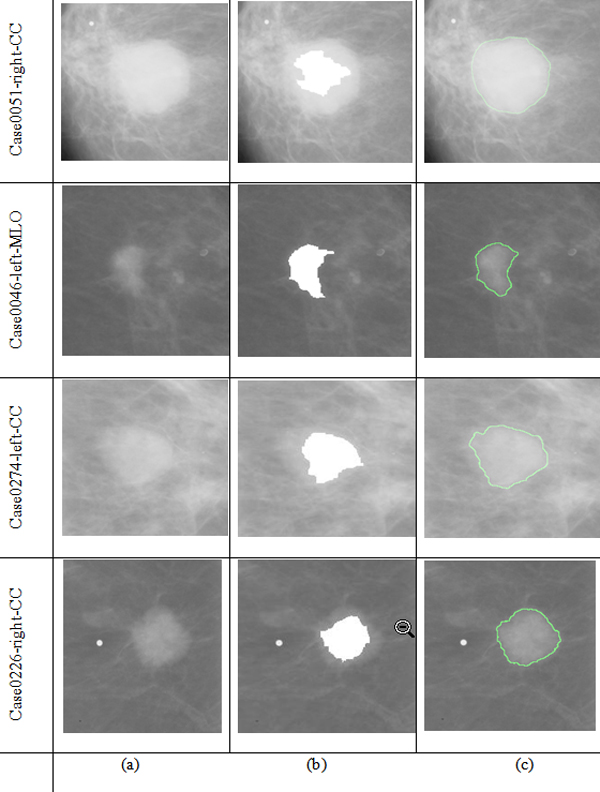
**(a) Original images selected from DDSM; (b) Markers and object boundaries superimposed using watershed algorithm on original images; (c) The final segment results based on improved level set**.

A program was developed using Matlab to run on all the test images without user intervention. The results show that all cases of segmentation were accurate in comparison with the radiologist-marked on the mammograms. Figure 1 shows some mammograms from DDSM and the segmentation results using watershed transform and level set based segmentation method.

### Segmentation evaluation

**Figure 2:**
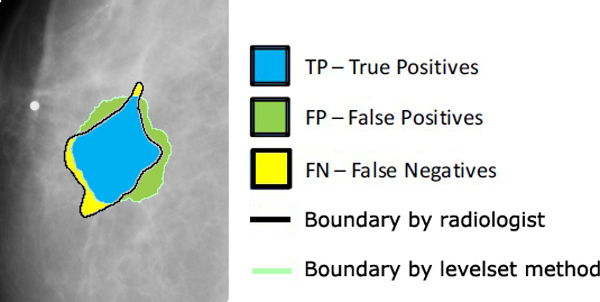
**True Positives, False Positives, and False Negatives definition**.

In the past, there have proposed many segmentation evaluation methods, however, segmentation evaluation is still an open topic [25,26]. There are mainly two evaluation methods. One is subjective evaluation, the other is objective evaluation. In subjective evaluation, visual check is often adopted while the segmentation obtained by the computer is evaluated against the segmentation obtained by a technician in objective evaluation. In this paper, we adopt objective evaluation. The evaluation measures used in the paper are [25]:

$$Hitting=\frac{TP}{TP+FN}$$

$$\text{Missing}=\frac{FN}{TP+FN}$$

$$OverHitting=\frac{FP}{TP+FN}$$

$$\text{Re}\;lativeHitting=\frac{TP}{TP+FP}$$

$$\text{Re}\;lativeMis\text{s}\;ing=\frac{FN}{TP+FP}$$

$$Kapps=\frac{2*Hitting}{2*Hitting+Mis\sin g+OverHitting}$$

where TP, FP and FN are True Positives, False Positives, and False Negatives respectively. Figure 2 shows the basic idea of TP, FP and FN of a mass segmentation. In Figure 2, TP represents the intersection of the radiologist and the algorithm, FP represents the segmentation results obtained only by the algorithm and the FN represents the segmentation results obtained only by the radiologist [25]. *Hitting *denotes the ratio of correct segmentation, *Missing *denotes the ratio of missing mass, *OverHitting *denotes the ratio of false mass segmented, *RelativeHitting *denotes relative correct ratio against segmentation results, and *RelativeMissing *denotes relative missing ratio against segmentation results [25].

### Segmentation results

**Figure 3:**
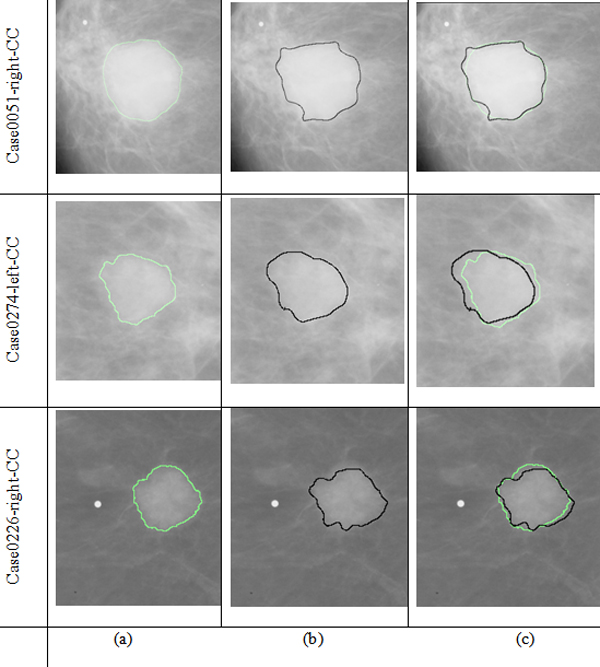
**Flowchart of the result of segmentation algorithm**. (a)The final segment results based on improved level set; (b) The region marked by the radiologist; (c) The Comparison between (a) and (b).

The comparisons of the segmentation results between the proposed method and the manually segmented image by radiologist are shown in Figure 3. In Figure 3, the black contours are the segmentation results using the proposed algorithm and the green contours are the results obtained by a radiologist. From Figure 3, we can find that the proposed method can obtain good results. We can find that the contours obtained by the proposed algorithm are closed to the contours obtained by the radiologist and it proves that the proposed algorithm is effective. Table 1 and Table 2 show the results of quantitative analysis and from the results we can also prove the effectiveness of the proposed algorithm.

**Table 1:** The different part Data (pixels) of Fig.3

*CaseNo*	*TP*	*FP*	*FN*
0046	4517	635	825
0051	3235	370	179
0069	2913	1475	140
0074	12912	2611	4654
0123	7419	1452	2566
0161	4339	2050	858
0226	18834	890	575
0274	1583	704	80

**Table 2:** Validation measure Data (percent) of Fig 3

*CaseNo*	*Hitting*	*Missing*	*OverHitting*	*RelativeHitting*	*RelativeMissing*	*Kappa*
0046	0.85	0.15	0.12	0.88	0.16	0.86
0051	0.95	0.05	0.11	0.90	0.05	0.92
0069	0.95	0.05	0.48	0.66	0.03	0.78
0074	0.74	0.26	0.15	0.83	0.30	0.78
0123	0.74	0.26	0.15	0.84	0.29	0.79
0161	0.83	0.17	0.39	0.68	0.13	0.75
0226	0.97	0.03	0.05	0.95	0.03	0.96
0274	0.95	0.05	0.42	0.69	0.03	0.80

**Figure 4:**
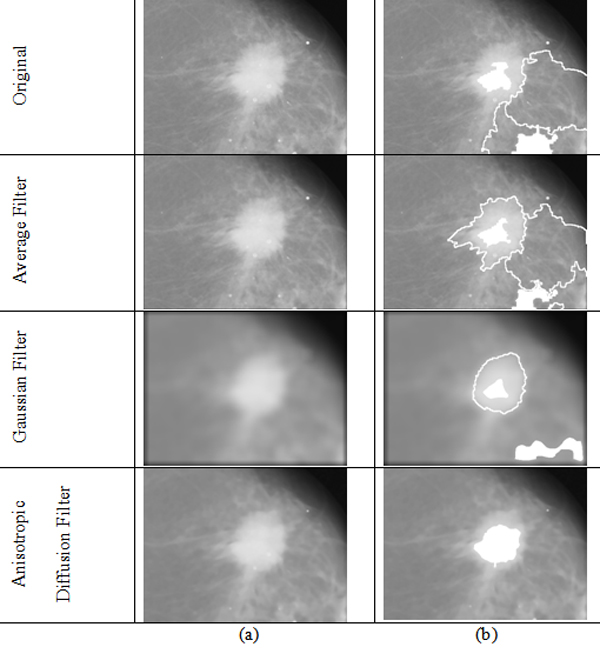
**(a) The result after different filter; (b) The segment results based on (a)**.

Besides the comparison of the proposed algorithm with the human segmentation, we also compared the effectiveness of different noise reduction technologies for over-segmentation reduction. The comparison results are shown in Figure 4. From Figure 4, we can find that effectiveness of average filter is worse than Gaussian filter while Gaussian filter is worse than anisotropic diffusion filter. Anisotropic diffusion filter can reduce the over-segmentation effectively and thus in the proposed algorithm we adopted anisotropic diffusion filter.

## Discussion

In this paper, we propose a mass segmentation algorithm which combines watershed method and level set method. The new method is divided into two steps: a marker-controlled watershed transform is first used to segment the mass region roughly, and then a level set is used to refine the segmentation.

Watershed based segmentation algorithm has many advantages which can overcome the disadvantage in the level set based segmentation. As we know, level set method usually needs hundreds of iterations to get a good segmentation result. With a good initialization provided by watershed segmentation, the level set method can converge more quickly, thus greatly speed up the whole segmentation procedure. Besides, by using watershed segmentation as the initialization step, we can remove the manual initialization step in general level set segmentation and we can obtain a full automatic segmentation algorithm.

However, the proposed algorithm still has a few limitations. In the proposed algorithm, the object to be segmented is already ROI images which have been preliminarily cut from the whole mammograms. Thus a mass detection step needs to be merged into the algorithm in the future. Although Noise reduction technologies are introduced into the algorithms, over-segmentation still happens on some mammographic images. Over-segmentation affects the efficiency of the algorithm and thus an effective over-segmentation algorithm is needed in the future. Another issue is the time complexity of the level set. By using the result from watershed we can save a lot time but much longer computation time is still needed to achieve the accurate segmentation results.

## Conclusions

In this paper, we have developed a hybrid method to segment the mammograms which used watershed algorithm and level set method. We used watershed transform to provide a coarse and fast pre-segmentation, and used the resultant segmentation as the initial contour for the level set segmentation. Automatic selection of the starting point from watershed transform can reduce the user interaction. The combination of the two segmentation methods speeds up the entire segmentation processing and improves the segmentation efficiency. Besides, the method has good topological adaptability; it can deal with complex and changing shapes of the segmentation of the mammograms well and get high segmentation accuracy. Experimental results show that the proposed segmentation method can obtain good results.

## Method

Mass segmentation includes two steps in the proposed algorithm. The first step is to use watershed transform for rough segmentation and the second step is to use level set based method to refine the segmentation obtained by watershed transform. Watershed based algorithms are mathematical morphology methods for image segmentation and they have many advantages in comparison with other image segmentation methods. For example, watershed transform based segmentation methods generally have high computation speed and can obtain closed contour lines and accurate position. Besides, watershed based image segmentation algorithms can handle weak edges very well [27].

**Figure 5:**
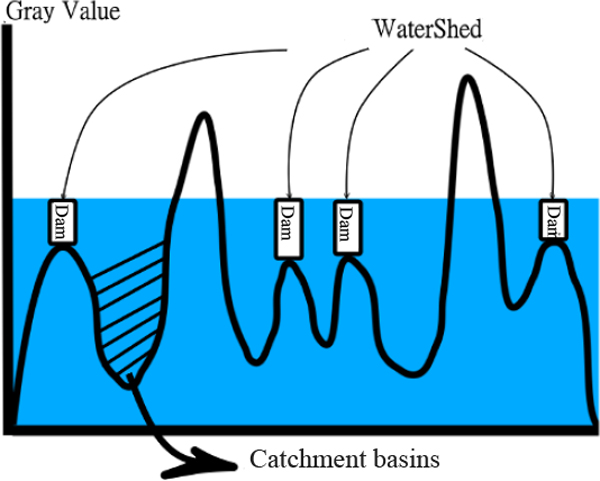
**Watershed**.

The basic idea of watershed can be described as follows [27]: let *χ *be a gray image, ||∇*χ*|| is the gradient image obtained from *χ*. In order to segment the objects in the image, the foreground markers will be computed for the objects. After the markers are obtained, the flood waves will propagate from the set of markers to cover the topographic surface ||∇*χ*|| [27]. When the water reaches the maximum gray value, the edges of the union of all dams come into being the watershed segmentation. Figure 5 shows the definition of watershed.

**Figure 6:**
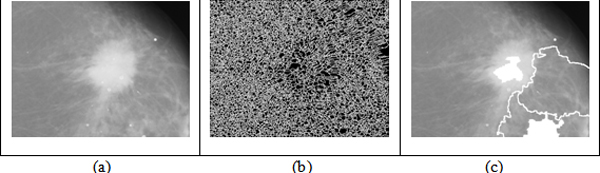
**(a) Original image; (b) Gradient based watershed method; (c) Marker based watershed**.

In the implementation of the watershed algorithm, if we only use gradient of watershed for segmentation, there are too many ridgelines which will cause over-segmentation (see Figure 6(b)). In order to reduce the over-segmentation, marker-controller watershed is used to reduce over-segmentation. In mark based watershed method, markers are connected through the component. After the marker-based watershed applied, we can get Figure 6(c).

After the image is segmented using watershed transform, we will use the resultant contour as the initial contour for a level set based method to refine the segmentation. The level set algorithm used for the segmentation in the proposed algorithm is from [28]. The level set algorithm proposed in [28] is based on region based active contour model. This model assumes an image is formed by two homogeneous regions, and can be formulated by the following energy functional [29,30]:

(1) $$E^{CV}\left(C,c_{1},c_{2}\right)=\lambda_{1}\int_{inside\left(C\right)}{\left|I_{0}\left(x,y\right)-c_{1}\right|}^{2}dxdy+\lambda_{2}\int_{outside\left(C\right)}{\left|I_{0}\left(x,y\right)-c_{2}\right|}^{2}dxdy+\mu \left|C\right|\left(\begin{array}{cc} \lambda_{1},\lambda_{2}\geq 0, & \mu \geq 0 \end{array}\right)$$

Where *λ*_1_, *λ*_1_, *μ*, *c*_1_, *c*_2 _are constants,*C *is the evolving contour, |*C*| is the length of contour *C*, *inside*(*C*) and *outside*(*C*) are the regions inside and outside the contour.

Although the proposed level set method could produce successful segmentation, it needs powerful initialization techniques. In order to solve the problem, in the proposed method, we use the contour obtained from watershed segmentation step as the initial contour of the level set. We resolve the drawbacks of the two methods mentioned above by combining them.

Besides the initialization issue, there is also noise issue. In general, the mammograms have a lot of noise. If the watershed algorithm was applied on the image directly, over-segmentation will happen because the watershed algorithm is very sensitive to noise. To avoid over-segmentation, we need to remove the noise. When the noise is removed, we can get the coarse segmentation using watersheds. The noise reduction methods investigated in the proposed paper include average filter, Gaussian filter and anisotropic diffusion [31]. Anisotropic diffusion was introduced by Perona and Malik [31] and it uses the gradient between the image area to control diffusion degree. Anisotropic diffusion can eliminate the noise effectively while preserve the edge of the image. The anisotropic diffusion used in the proposed algorithm is the method developed in the [32].

**Figure 7:**
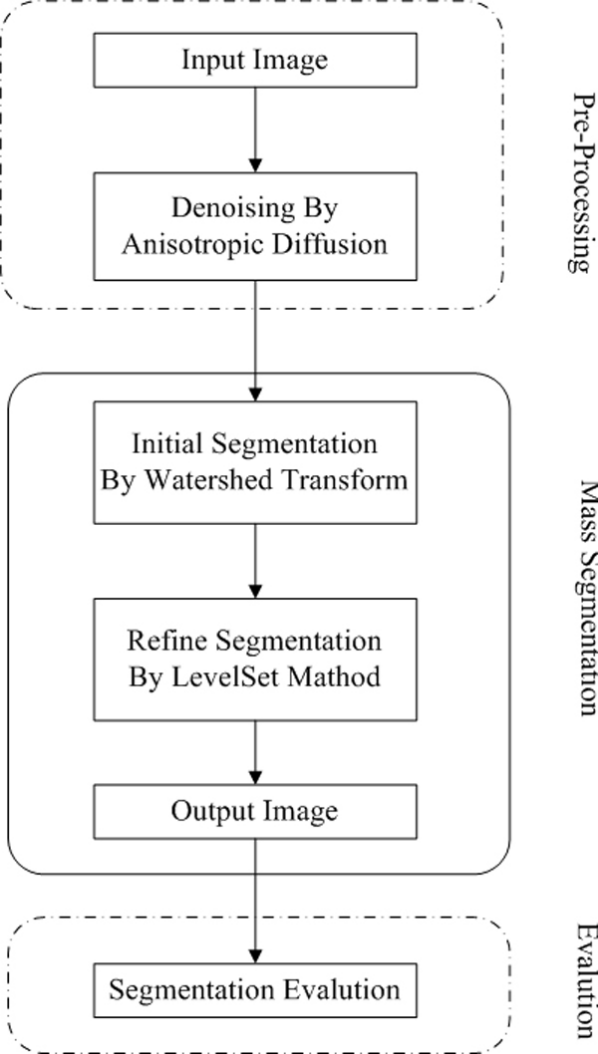
**Flowchart of the segmentation algorithm**.

The proposed algorithm is shown in Figure 7. It is composed of several steps, the original image will be preprocessed and then used as the input of the watershed segmentation and the rough segmentation is obtained. The rough segmentation will be used as the start contour for the level set segmentation. This approach combines the advantages of the two methods and overcome the disadvantages of each single method: marker-based watershed is rough but fast and the level set segmentation needs a certain number of iterations, which produces the final, highly accurate, smooth results.

## Competing interests

The authors declare that they have no competing interests.

## Authors' contributions

JL, XL, LC and JC developed the algorithm using watershed and level let and wrote the original the paper. JT proposed the investigation of over-segmentation issue and revised the paper. YD did data analysis. All authors read and approved the final manuscript.
